# Systemic treatment of a metastatic carotid body tumor: A case report and literature review

**DOI:** 10.1097/MD.0000000000022811

**Published:** 2020-11-20

**Authors:** Jiazhang Xing, Yuejuan Cheng, Hongyan Ying, Mei Guan, Ning Jia, Chunmei Bai

**Affiliations:** Department of Medical Oncology, Peking Union Medical College Hospital, Chinese Academy of Medical Sciences, Beijing, China.

**Keywords:** carotid body tumor, distant metastasis, paraganglioma, systemic therapy

## Abstract

**Rationale:**

Carotid body tumors (CBTs) are head and neck paragangliomas (PGLs) with a low incidence of distant metastasis. To date, only a few metastatic cases treated with detailed systemic therapy are reported and effective management is still inconclusive. Herein, we reported a metastatic CBT case with systemic therapy and reviewed the reported systemic treatment.

**Patient concerns:**

A 56-year-old man noticed multiple painless nodules on the right side of the neck and developed debilitating chest and back pain 7 years after the CBT resection.

**Diagnoses:**

Widespread bone and lymph nodes CBT metastases.

**Interventions:**

Biopsies of the enlarged lymph nodes confirmed the diagnosis of metastatic CBT and 18F-FDG PET-CT detected multiple right cervical lymph nodes and bone metastases. 24 cycles of cyclophosphamide, vincristine and dacarbazine (CVD) chemotherapy were given since May 2016 to Jul 2018 and dacarbazine maintenance therapy was given in the next 15 months follow-up period.

**Outcomes:**

Partial remission was achieved according to the Response Evaluation in Criteria in Solid Tumors 1.1 criteria. A prominent control in the metastatic lesions were also observed in 18F-FDG PET-CT scan.

**Lessons:**

Evidence for systemic management of metastatic CBTs is mainly based on studies of PGLs and pheochromocytoma. According to our review on metastatic CBT cases treated with systemic therapy from 1981 to 2018, chemotherapy, especially the CVD regimen, was a common reported management. In SDHB mutated patients, sunitinib and temozolomide could also be considered.

## 1 Introduction

Paragangliomas (PGLs) are a subgroup of neuroendocrine tumors (NETs) arising from the paraganglia, which is a collection of cells from the embryonic nervous tissue. Head and neck PGLs are rare, comprising 0.03% of all tumors.^[1]^ A carotid body tumor (CBT) is the most common head and neck PGL. It grows slowly and originates from the carotid body chemoreceptors. Distant metastasis of CBT is rare. Up to now, approximately 1200 CBT cases have been reported and few cases have been associated with distant metastases.^[2]^ Owing to the low incidence of metastatic CBT, the specific systemic management for metastatic CBT is inconclusive. Most patients were given the same regimen as that used for PGLs originating from other sites.

Here, we report a case of a 56-year-old man with multiple bone and cervical lymph node metastases from a CBT who had both symptomatic and oncological remission after chemotherapy. We also performed a literature review of the systemic treatment of metastatic CBT.

## 2 Case report

**Figure 1:**
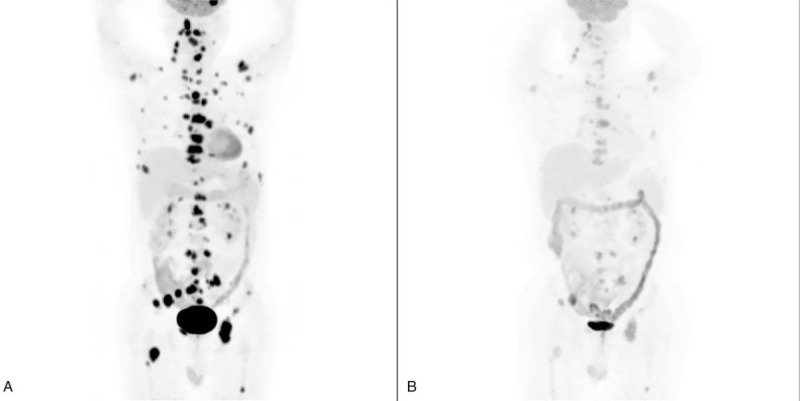
(A, B) The ^18^F-FDG PET-CT of the whole body of a 56-year-old man who suffered from CBT metastasis before (A) and after (B) treatment with combined chemotherapy, which consisted of CVD. Before the CVD regimen, multiple right cervical lymph nodes and bone (skull, vertebrae, sternum, clavicles, humeri, scapulae, ribs, pelvis, femora) metastases were detected (A). Prominent decreased uptake in the metastatic lymph nodes and bone lesions was observed after CVD treatment. (B).

The patient was a 56-year-old man who noticed a slow-growing mass on the right neck in 2003. In 2009, a computerized tomography angiogram (CTA) revealed a tumor located at the bifurcation of the right carotid artery, and CBT was suspected. Tumor resection with grafting of the carotid artery using a great saphenous vein graft and lymphadenectomy was performed in July 2009. During the surgical exploration, the tumor was measured at 6 cm × 5 cm, enclosed the internal and external carotid arteries, and was receiving blood from the external carotid artery. Postoperative pathology was consistent with a CBT without lymph node metastasis. The tumor cells were immunohistochemically positive for synaptophysin, chromogranin A and S-100, and the ki67 index was 1%. He was asymptomatic until February 2016, when multiple painless nodules were found on the right side of the neck. A neck CTA revealed multiple enlarged cervical lymph nodes and biopsies of the enlarged lymph nodes confirmed the diagnosis of metastatic CBT with a ki67 index of 5%. The patient also developed debilitating chest and back pain, and analgesics were prescribed to relieve the pain. A ^18^F-fluorodeoxyglucose positron emission tomography (^18^F-FDG PET) CT scan detected multiple right cervical lymph nodes and bone metastases (skull, vertebrae, sternum, clavicles, humeri, scapulae, ribs, pelvis, femora) (Fig. 1A), while the 68Ga-DOTA-TATE PET-CT scan was negative. Although the patient had no known family history of CBT, a germline mutation test showed a succinate dehydrogenase subunit B (SDHB) gene mutation.

In May 2016, the first cycle of chemotherapy, consisting of cyclophosphamide, vincristine and dacarbazine (CVD), was carried out. Zoledronic acid was also given. In July 2018, when the chemotherapy was stopped, the patient received 24 cycles of CVD chemotherapy. He tolerated chemotherapy well with grade 2 nausea/vomiting, grade 2 leukopenia and grade 1 neutropenia. Before chemotherapy was initiated, his hemoglobin was 63 g/l, while his white blood cells and platelets were within the normal range. His anemia was considered cancer-related, and a red blood cell transfusion was given with the initial 3 cycles of chemotherapy. His hemoglobin was stabilized at 80 to 90 g/l during subsequent chemotherapy sessions without red blood cell transfusion and then recovered to normal levels when chemotherapy ceased. The patient's pain resolved after 3 cycles of chemotherapy. The best radiologic response to therapy using the Response Evaluation in Criteria in Solid Tumors (RECIST) 1.1 criteria was partial remission. A repeated ^18^F-FDG PET-CT scan also demonstrated a prominent decreased uptake in the metastatic lymph nodes and bone lesions (Fig. 1B). He gained 15 kg since chemotherapy was initiated. During the 15 months follow-up, the patient was in dacarbazine maintenance therapy and was tolerated well without tumor progression.

## 3 Discussion

CBT is a non-secreting PGL without prominent clinical manifestations. The incidence of metastasis is approximately 5% to 10%, while distant metastasis is rare.^[3]^ Surgery is the curative treatment for localized CBT, and the appropriate operative method and postsurgical treatment options are still being debated. Due to the rarity of distant metastasis, the optimal treatment strategies for metastatic diseases remain to be identified. The choice of appropriate systemic management is challenging and most evidence is based on small-scale retrospective studies of PGLs and pheochromocytoma (PCCs). Although metastatic CBT cases have been reviewed previously,^[2]^ the specific response to systemic therapy was not mentioned in the study. Thus, we searched Medline articles published from 1981 to 2018 for systemic therapy for distant metastatic CBT. Data from 10 patients were reviewed,^[4–10]^ and the patient characteristics, responses to local and systemic treatments and patient survival are summarized in Table 1.

**Table 1:** Review of systemic therapies for CBTs with distant metastases (1981–2018).

Case	Publish year	Age (yr)	Gender	Time to metastasis (yr)	Metastatic sites	Mutation	Systemic treatment	Response	Survival after diagnosis (yr)	Survival after metastasis (yr)
Massey and Wallner^[4]^	1992	21	F	2	Retroperitoneum	unclear	CTX/VCR/DOX	PD	10	8
Massey and Wallner^[4]^	1992	26	M	13	Bones	unclear	CTX/VCR/DOX (3 cycles)	PD	22	9
Patel et al^[5]^	1995	46	F	NA	Bones, lung	unclear	DOX/DTIC	PD	NA	AWD/8
							Ifosfamide	PD		
							Strontium 89	SD		
Patel et al^[5]^	1995	67	F	NA	Bones	unclear	Etoposide/DDP	SD	NA	AWD/5
							CVD/DOX	SD		
Hajnžić et al^[6]^	1999	7	M	2	Lung	unclear	CTX/VCR/DOX	PD	2	4 months
Pacheco-Ojeda^[7]^	2001	40	M	8	Lung	unclear	CTX/DOX/DDP (2 cycles)	SD	10	2
							Interferon	PD		
Havekes et al^[8]^	2007	28	F	30	Abdomen	SDHD	Octreotide	PD	32	2
Jeevan et al^[9]^	2016	77	F	Synchronous	Bones	unclear	Sunitinib	SD	2	2
Kumari et al ^[10]^	2017	47	F	Synchronous	Lung, bones	unclear	CVD (3 cycles)	SD	NA	NA
							PRRT	PD		
Case	2019	56	M	7	Bones	SDHB	CVD (24 cycles)	PR	NA	AWD/2

AWD/ = alive with disease/followed years, CTX = cyclophosphamide, CVD = cyclophosphamide, vincristine, dacarbazine, DDP = cisplatin, DOX = doxorubicin, DTIC = dacarbazine, NA = not available, PD = progressive disease, PR = partial response, PRRT = peptide receptor radionuclide therapy, SD = stable disease, VCR = vincristine.

The patients’ ages ranged from 7 to 77 years. There were 4 males and 6 females. The metastatic sites included the lungs, bones, abdomen, and retroperitoneum. In this review, the time to development of distant metastases varied from 0 to 30 years after diagnosis. The median and average time to distant metastasis were 7.5 and 10.3 years after diagnosis, respectively.

Among the 10 patients, combination chemotherapy was most commonly used, although the regimens varied. Two patients receiving the CVD regimen achieved stable and partial disease response, respectively. Three patients using another chemotherapy regimen consisting of cyclophosphamide, vincristine and doxorubicin all had disease progression. CVD is the most commonly used regimen for malignant PCCs/PGLs. The patient we reported received CVD for 24 cycles, and a partial response was achieved with tolerable side effects. In a 22-year follow-up study of 18 patients with malignant PCC/PGL treated with CVD, 2 patients (11%) had a complete response, and 8 patients (44%) had a partial response.^[11]^ Two other studies observed that 47.1% and 26% of malignant PCC/PGL patients showed biochemical or tumor responses to CVD, respectively.^[12,13]^ Temozolomide, an alternative medicine to dacarbazine, has been used as a monotherapy for malignant PCC/PGL in recent years. In a study of 15 malignant PCC/PGL patients who received temozolomide, a partial response was observed in 33% of the patients and the effectiveness of temozolomide seemed to be related to the SDHB mutation. Partial responses were only observed in patients with SDHB mutations, and the progression-free survival was statistically longer than that in patients without the mutation.^[14]^

Mutations in SDHB, SDHC and SDHD were shown to be associated with overexpression in angiogenesis and in angiogenic molecules (including vascular endothelial growth factor (VEGF) and VEGF receptors) in PCCs/PGLs.^[15,16]^ Thus, some antiangiogenic agents have been proposed for PGLs therapy. Sunitinib has been shown to be effective for malignant PGLs, especially in carriers of the SDHB mutation.^[17]^ In a recent review including all cases of malignant PGL treated with sunitinib therapy, sunitinib was demonstrated to be effective in 72.2% of the 36 patients.^[18]^ In our review, 1 patient that received sunitinib for malignant CBT management achieved disease stabilization.

For malignant PCCs/PGLs, radioactive ^131^I-meta-iodobenzylguanidine (^131^I-MIBG) therapy was also an effective method with an estimated response rate of 30%,^[19]^ but ^131^I-MIBG was rarely used in CBTs. The efficacy of peptide receptor radioligand therapy (PRRT) using ^90^Y-dotatoc and ^177^Lu-DOTATATE for malignant PCCs/PGLs has also been described in isolated case reports and small series.^[20,21]^ One patient with lung and bone metastases in our review experienced disease progression after treatment with PRRT.

For systemic management of distant metastatic CBTs, most evidence is based on studies on PGLs or PCCs. Because of the rarity of CBTs and their different clinicopathological features, the efficacy of active systemic treatment in PCCs/PGLs still needs to be determined in metastatic CBTs. By our case series review, chemotherapy, especially the CVD regimen, was the most common reported therapy for metastatic CBTs. Sunitinib also showed efficacy in disease control, especially in patients with SDHB mutation.

## 4 Conclusion

Although distant metastasis of CBT is rare, long-term follow-up is necessary because of the possibility of disease recurrence many years after primary tumor resection. For the systemic management of disseminated metastatic CBT, most evidence is based on studies on PGLs or PCCs. In our case series, chemotherapy, especially the CVD regimen, and sunitinib were used for metastatic CBT and showed efficacy in disease control. Effective treatment for malignant PCCs/PGLs might be used for metastatic CBT, although larger cohort studies are warranted.

## Author contributions

**Conceptualization:** Jiazhang Xing, Yuejuan Cheng, Hongyan Ying.

**Data curation:** Jiazhang Xing, Yuejuan Cheng, Mei Guan, Ning Jia.

**Formal analysis:** Yuejuan Cheng, Mei Guan, Ning Jia.

**Funding acquisition:** Yuejuan Cheng, Hongyan Ying.

**Investigation:** Jiazhang Xing, Hongyan Ying.

**Software:** Jiazhang Xing.

**Supervision:** Hongyan Ying, Mei Guan, Ning Jia, Chunmei Bai.

**Validation:** Hongyan Ying, Chunmei Bai.

**Writing – original draft:** Jiazhang Xing, Yuejuan Cheng.

**Writing – review & editing:** Hongyan Ying, Mei Guan, Ning Jia, Chunmei Bai.
